# Risk factors for post-ERCP cholecystitis: a single-center retrospective study

**DOI:** 10.1186/s12876-018-0854-3

**Published:** 2018-08-22

**Authors:** Jun Cao, Chunyan Peng, Xiwei Ding, Yonghua Shen, Han Wu, Ruhua Zheng, Lei Wang, Xiaoping Zou

**Affiliations:** 1 Department of Gastroenterology, Nanjing Drum Tower Hospital, The Affiliated Hospital of Nanjing University Medical School, Nanjing, China; 2 Zhongshan Road 321, Department of Gastroenterology, Nanjing Drum Tower Hospital, The Affiliated Hospital of Nanjing University Medical School, Nanjing, 210008 Jiang Su Province China

**Keywords:** ERCP, Cholecystitis, Nomogram, Risk factors, Success prediction

## Abstract

**Background:**

The risk factors for post-ERCP cholecystitis (PEC) have not been characterized. Hence, this study aimed to identify the potential risk factors for PEC.

**Methods:**

The medical records of 4238 patients undergoing the first ERCP in a single center from January 2012 to December 2016 were analyzed in this study. A multivariate analysis was used to identify the risk factors.

**Results:**

This study included 2672 patients who met the enrollment criteria. Of these, 36 patients (incidence rate of 1.35%) developed PEC within 2 weeks of the procedure. Univariate and multivariate analyses identified the following factors associated with PEC: history of acute pancreatitis [odds ratio (OR) = 2.60; 95% confidence interval (CI): 1.29–5.23], history of chronic cholecystitis (OR = 8.47; 95% CI: 2.54–28.24), gallbladder opacification (OR = 2.79; 95% CI: 1.37–5.70), biliary duct metallic stent placement (OR = 3.66; 95% CI: 1.78–7.54), and high leukocyte count before ERCP (OR = 1.10; 95% CI: 1.04–1.17). The prediction model incorporating these factors demonstrated an area under the receiver operating characteristic curve of 0.85 (95% CI, 0.80–0.91). A prognostic nomogram was developed using the aforementioned variables to estimate the probability of PEC.

**Conclusions:**

The risk factors, including the history of acute pancreatitis, history of chronic cholecystitis, gallbladder opacification, biliary duct metallic stent placement, and high leucocyte counts before ERCP, increased the occurrence of PEC and were positive predictors for PEC. The constructed nomogram was used to estimate the risk of PEC, guiding the implementation of prophylactic measures to prevent PEC in clinical practice.

## Background

Endoscopic retrograde cholangiopancreatography (ERCP) is an endoscopic procedure performed under visual and fluoroscopic guidance. It is widely used in diagnosing and treating of biliary and pancreatic diseases. ERCP is a technically challenging endoscopic procedure that can cause serious adverse events and occasionally even death. Possible ERCP-related adverse events include acute pancreatitis, hemorrhage, perforation, cholangitis, and acute cholecystitis. Of these, post-ERCP pancreatitis (PEP) is the most common one with 9.7% incidence and 0.7% mortality rate [1]. Due to its high incidence, numerous studies have investigated the risk factors of PEP. The risk factors of PEP include suspected sphincter of Oddi dysfunction, major papilla pancreatogram, needle-knife precut, and female gender [2, 3].

In contrast, post-ERCP cholecystitis (PEC) gained much less attention. Freemen et al. reported cholecystitis in 0.5% (11/2347) of patients 16 days after biliary sphincterotomy [4]. In this study, no predictors of cholecystitis were identified other than the presence of stones in the gallbladder. Most studies reporting the adverse events of ERCP did not investigate the risk factors and predictors of PEC alone, which might be due to its relatively low incidence. However, most PECs require emergency cholecystectomy and extended hospitalization time. In addition, some PECs are severe and potentially fatal. Identifying the risk factors for PEC may help prevent this adverse event. The aim of this study was to assess the risk factors for PEC in patients with gallbladder in situ within 2 weeks of procedure in a single large-volume center.

## Methods

The study was approved by the Ethical Committee at Nanjing Drum Tower Hospital Affiliated to Nanjing University Medical School (study number 2017–167-01). All subjects were anonymized; hence, informed consent was not required. This study conformed to Strengthening the Reporting of Observational Studies in Epidemiology guidelines.

### Patients

The medical records of patients with gallbladder in situ who underwent ERCP for the first time in the hospital from January 2012 to December 2016 were reviewed and analyzed retrospectively. Patients who had concomitant acute cholecystitis at the time of ERCP or had a previous ERCP history were excluded from the study. The medical records of eligible patients were reviewed retrospectively to identify any occurrence of acute cholecystitis within 2 weeks after ERCP.

### Risk factors

The following predefined parameters were analyzed for PEC within 2 weeks. The demographic information included the following: age and sex. The past history included the following: acute pancreatitis, chronic cholecystitis, acute cholangitis, hypertension, hyperlipidemia, and diabetes mellitus. The laboratory examination indexes before ERCP were as follows: alanine aminotransferase, aspartate aminotransferase, alkaline phosphatase, gamma-glutamyltranspeptidase, total bilirubin, direct bilirubin, leukocyte count, hemoglobin, and platelet count. The indexes during ERCP were as follows: gallbladder opacification, biliary duct stent, and common bile duct (CBD) diameter. Other factors before ERCP included temperature and antibiotics. During the bile duct opacification, we recorded gallbladder opacification if contrast medium entered into gallbladder and the outline of gallbladder could be seen. No additional efforts were made to get the entire gallbladder outlined by contrast medium if this had not been accomplished simultaneously with bile duct opacification.

### Endoscopy protocol

Duodenal side-viewing endoscopes (JF-260, TJF-240, or TJF-260; Olympus, Tokyo, Japan) were used to perform the ERCP procedure. The patients were under midazolam sedation. Sphincterotomy was performed using a standard sphincterotome and/or a needle knife. Balloon sphincteroplasty was performed using a Boston Scientific controlled radial expansion balloon with a diameter range of 12–15 mm, 15–18 mm or 18–20 mm). Stones were extracted using retrieval baskets and/or balloon-tipped catheters. An endoscopic mechanical lithotripsy or laser lithotripsy was attempted to crush down the stones if the stones were too big to remove. Obstructive jaundice resulting from malignant bile duct stenosis was treated by placing nasobiliary drainage (ENBD), plastic stents, or self-expandable biliary metal stents. The benign biliary stricture was treated by dilation or placement of plastic stents or fully covered self-expandable biliary metal stents. Pancreaticobiliary maljunction or pancreas divisum was treated by placing ENBD or plastic stents.

### Diagnostic criteria of acute cholecystitis

Acute cholecystitis was diagnosed according to the 2018 Tokyo guidelines of acute cholecystitis [5]. The diagnostic criteria were based on the following three aspects: (A) local signs of inflammation, including (1) Murphy’s sign and (2) right upper abdominal quadrant mass/pain/tenderness; (B) systemic signs of inflammation, including (1) fever, (2) elevated CRP, and (3) elevated WBC count; and (C) imaging finding characteristic of acute cholecystitis. A definite diagnosis was as follows: one item in A + one item in B + C.

### Statistical analysis

Mean ± standard deviation was used to describe deviation of the data with the normal distribution of the variables. Median (quartile spacing) was used to describe the data that did not meet the normal distribution of the variables. Frequency (percentage) was used to describe the classification of variables. The differences between groups were compared using *t*-test, chi-square test, or rank-sum test [6]. Logistic regression was used to analyze the findings of a multivariate analysis of acute cholecystitis after ERCP [7]. The nomogram was used to visualize the logistic regression model [8]. The Bonferroni method was used to calibrate the adjusted test level for pairwise comparison of the findings of the chi-square test. Binned Scatterplot was used to describe the relationship between preoperative leukocytes and the risk of acute cholecystitis within 2 weeks after ERCP. SPSS 13.0 was used for statistical analysis. pROC and rms package in R 3.3.3 software were used to construct receiver operating characteristic (ROC) curve and nomogram. A two-tailed value of *P* <0.05 was established as the threshold of statistical significance.

## Results

### Patient population

**Fig. 1:**
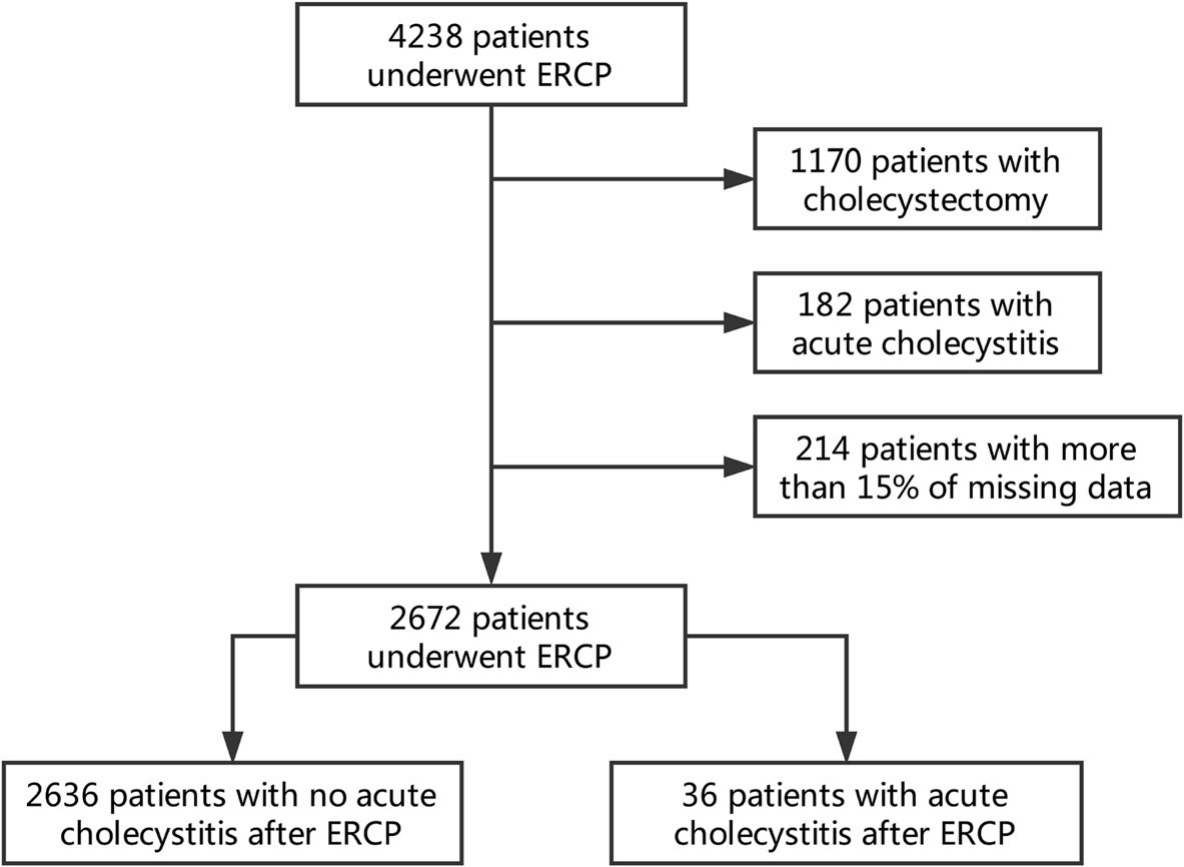
Flowchart of study results

A total of 4238 patients who underwent the first ERCP procedure between January 1, 2012, and December 31, 2016, in the hospital were included. Of these, 1352 patients were excluded from the study due to concomitant acute cholecystitis (*n* = 182) or a history of cholecystectomy (*n* = 1170) before ERCP. Further, 214 patients with more than 15% of missing data were also excluded. Finally, 2672 patients with intact gallbladder were included in the retrospective analysis to analyze the incidence of acute cholecystitis within 2 weeks after the initial ERCP. The mean age of the patients was 62.4 ± 16.2 years (range, 1–106 years); 1166 patients (43.6%) were female (Table 1). Also, 36 patients (incidence rate of 1.35%) finally developed acute cholecystitis within 2 weeks after the first ERCP (Fig. 1).

**Table 1:** Univariate analysis of potential risk factors for the development of acute cholecystitis after ERCP

Variable	Acute Cholecystitis		Statistic	*P*
	No (*n* = 2636)	Yes (*n* = 36)		
Age (y), Mean ± SD	62.4 ± 16.28	62.2 ± 12.90	*t* = 0.097	0.923
Female	1145 (43.4%)	21 (58.3%)	*x*^*2*^ = 2.627	0.105
Past history				
Hypertension	976 (37.0%)	13 (36.1%)	*x*^*2*^ = 0.013	0.910
Hyperlipemia	70 (2.7%)	1 (2.9%)	*x*^*2*^ = 0.005	0.942
Diabetes mellitus	473 (18.0%)	7 (20.0%)	*x*^*2*^ = 0.098	0.755
Acute pancreatitis	459 (17.4%)	16 (44.4%)	*x*^*2*^ = 17.754	< 0.001
Acute cholangitis	532 (20.2%)	6 (16.7%)	*x*^*2*^ = 0.273	0.601
Chronic cholecystitis	1427 (54.1%)	33 (91.7%)	*x*^*2*^ = 20.185	< 0.001
Antibiotics before ERCP	948 (36.0%)	11 (30.6%)	*x*^*2*^ = 0.451	0.601
Gallbladder opacification	1016 (38.5%)	24 (66.7%)	*x*^*2*^ = 11.816	0.001
Diameter of CBD(cm)	1.2 ± 0.48	1.1 ± 0.42	*t* = 1.237	0.216
Temperature before ERCP (°C)	36.6 ± 0.58	36.8 ± 0.84	*t* = −1.891	0.059
Bile duct stents			*x*^*2*^ = 15.805	0.001
No stent	1174 (66.2%)	19 (52.8%)		
Metallic stent	414 (15.7%)	14 (38.9%)		
Plastic stent	436 (16.5%)	2 (5.6%)		
Metallic+plastic stent	42 (1.6%)	1 (2.8%)		
Laboratory index before ERCP (Median,P_25_,P_75_)				
ALT	83.0 (37.3, 193.3)	58.7 (37.5, 191.0)	*Z* = −0.027	0.979
AST	54.9 (27.9, 119.0)	53.5 (28.7, 83.8)	*Z* = −0.143	0.886
AKP	198.1 (114.9, 359.8)	196.3 (94.2, 321.2)	*Z* = −0.414	0.679
GGT	292.5 (125.7, 550.2)	246.9 (91.9, 543.8)	*Z* = −0.181	0.856
TB	34.9 (15.5, 126.8)	23.7 (13.7, 83.3)	*Z* = −0.845	0.391
DB	21.2 (6.7, 98.8)	12.8 (7.1, 73.4)	*Z* = −0.641	0.522
WBC	6.1 (4.8, 8.2)	7.9 (5.9, 11.0)	*Z* = −3.610	< 0.001
Hemoglobin	125.0 (111.0, 136.0)	131.0 (121.0, 138.0)	*Z* = −1.413	0.158
Platelet count	192.0 (148.0, 245.0)	192.0 (146.0, 276.0)	*Z* = −0.210	0.833

*CBD* common bile duct, *ALT* alanine aminotransferase*, AST* aspartate anminotransferase*, AKP* alkaline phosphatase*, GGT* gamma-glutamyltranspeptidase*, TB* total bilirubin*, DB* direct bilirubin*, WBC* white blood cell

### Risk factors for acute cholecystitis within 2 weeks after the first ERCP

The results of univariate analysis of potential risk factors for the development of acute cholecystitis within 2 weeks after ERCP are shown in Table 1. The following parameters were found to be closely correlated with PEC in the univariate analysis: history of acute pancreatitis (*χ*^*2*^ = 17.754, *P* < 0.001), chronic cholecystitis (*χ*^*2*^ = 20.815, *P* < 0.001), gallbladder opacification (*χ*^*2*^ = 11.816, *P* = 0.001), bile duct stents (*χ*^*2*^ = 15.805, *P* = 0.001), leukocyte count before ERCP (*Z = − 3.610, P* < 0.001). The multiple logistic regression analysis identified the following variables significantly correlated with post-ERCP acute cholecystitis (Table 2): history of acute pancreatitis (OR = 2.60; 95% CI: 1.29–5.23; *P* = 0.007); history of chronic cholecystitis (OR = 8.47; 95% CI: 2.54–28.24; *P* = 0.001), gallbladder opacification (OR = 2.79; 95% CI: 1.37–5.70; *P* = 0.005), biliary duct metallic stent placement (OR = 3.66; 95% CI: 1.78–7.54; *P*<0.001) and leukocyte count before ERCP (OR = 1.10; 95% CI: 1.04–1.17; *P* = 0.001). In the 36 patients who developed PEC, 29 had gallstones.

**Table 2:** Multivariate logistic regression analysis of potential risk factors for subsequent post-ERCP cholecystitis

Variable	*B*	*S.E*	*P*	*OR (95% CI)*
WBC before ERCP	0.099	0.029	0.001	1.10 (1.04, 1.17)
History of acute pancreatitis	0.955	0.357	0.007	2.60 (1.29, 5.23)
History of chronic cholecystitis	2.137	0.614	0.001	8.47 (2.54, 28.24)
Gallbladder opacification	1.026	0.364	0.005	2.79 (1.37, 5.70)
Stent types	–	–	0.001	–
No	reference	reference	reference	reference
Metallic stent	1.298	0.369	< 0.001	3.66 (1.78, 7.54)
Plastic stent	−0.578	0.759	0.446	0.56 (0.13, 2.48)
Metallic +plastic stent	1.735	1.077	0.107	5.67 (0.69, 46.78)
Constant	reference	reference	reference	reference

**Fig. 2:**
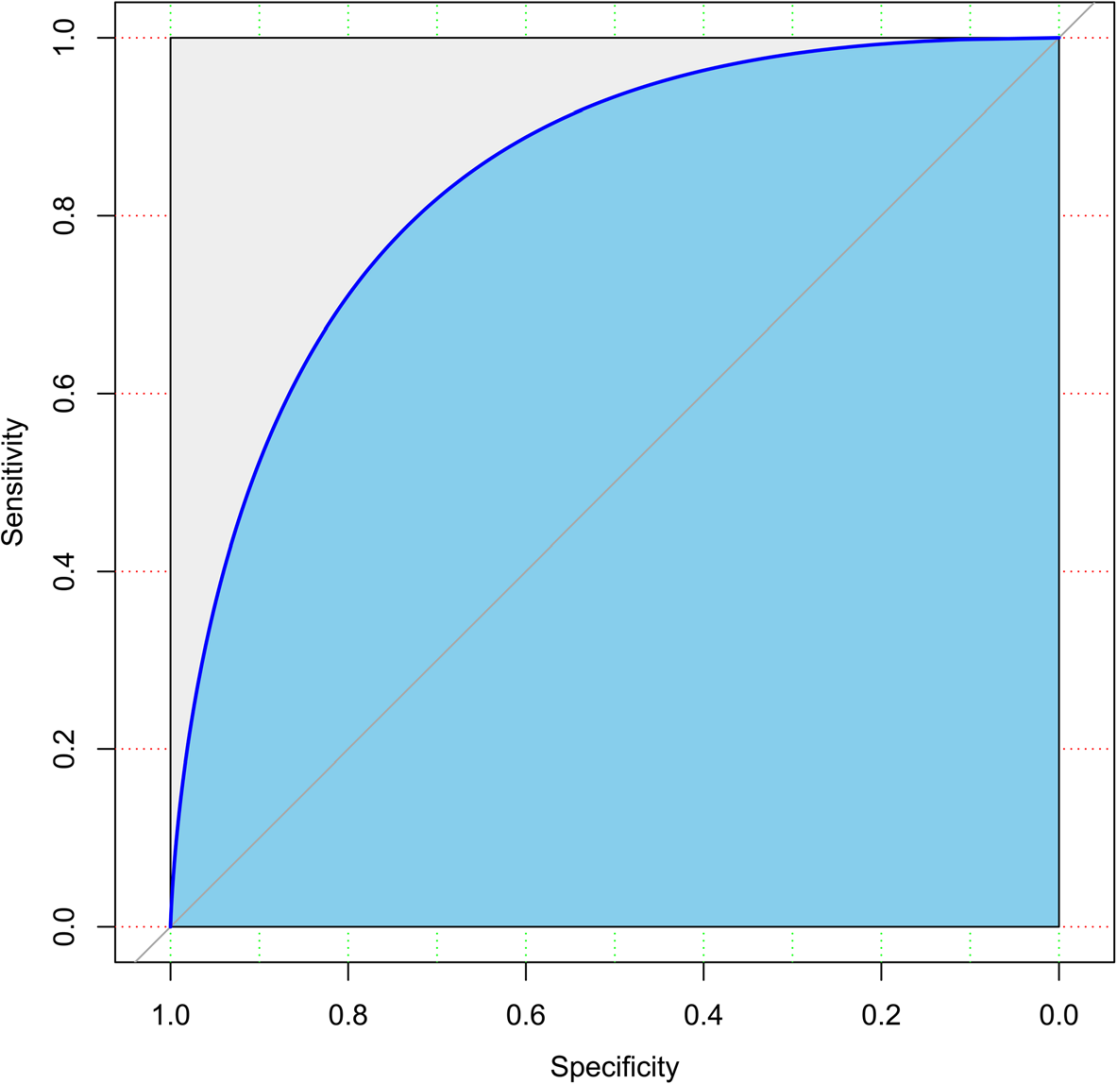
ROC curve for logistic regression model predicting post-ERCP cholecystitis. It included a history of chronic cholecystitis, history of pancreatitis, gallbladder opacification, leukocyte count, and biliary metallic duct stent. AUC = 0.85; 95% CI: 0.80–0.91

The multivariate models were built to predict the incidence of acute cholecystitis after ERCP within 2 weeks. According to the ROC of the multivariate model, the area under the curve (AUC) was 0.852; the sensitivity and specificity were 82.3% and 73.3%, respectively (Fig. 2). The result revealed a good concordance and a good predictive ability.

**Fig. 3:**
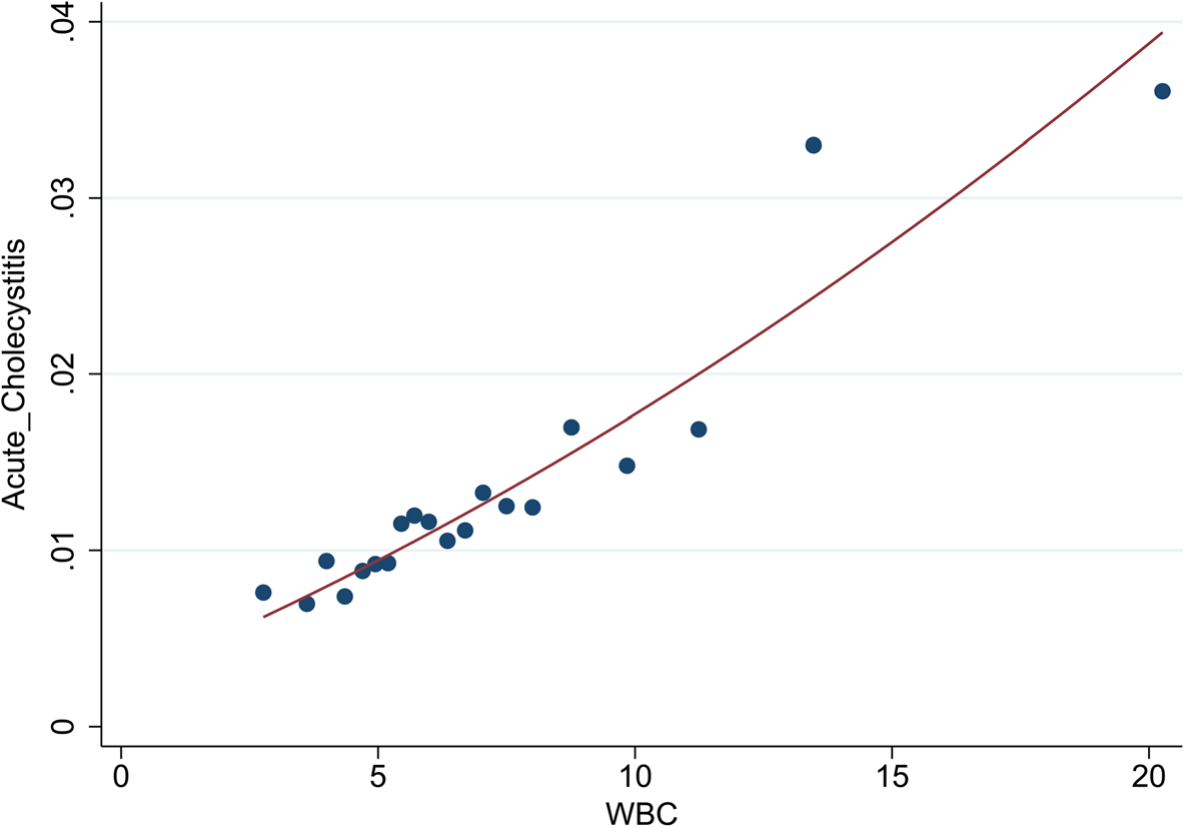
Binned scatterplot diagram of the relationship between leukocyte count before ERCP and the risk of post-ERCP cholecystitis

Finally, the correlation between white blood cell counts before ERCP and PEC was estimated using a binned scatterplot diagram (Fig. 3). The results indicated a curvilinear relationship; also, the risk of PEC increased with the increase in preoperative WBC.

## Discussion

Although PEC is not as common as PEP, it can lead to purulent cholecystitis and result in emergency operation or percutaneous transhepatic gallbladder drainage. Therefore, PEC should be recognized early. The present study, included 2666 patients with intact gallbladder who underwent the first ERCP, and the incidence of acute cholecystitis was 1.35% (36/2672) within 2 weeks after ERCP. The univariate and multivariate analyses indicated that the history of chronic cholecystitis, previous acute pancreatitis, gallbladder opacification, biliary stent placement, and high leukocyte count before ERCP were risk factors for the occurrence of PEC within 2 weeks of the procedure. Of note, biliary metallic stent placement significantly increased the occurrence of PEC.

As a risk factor for PEC, chronic cholecystitis may increase PEC perhaps owing to gallbladder contamination by nonsterile contrast or intestinal reflux. The diameter of the biliary duct metallic stent was greater than that of the plastic stent. Therefore, metallic stent placement during ERCP greatly increased duodenal biliary reflux, further increasing the possibility of PEC. Obstructions of the cystic duct by the stent may also contribute to the development of PEC. An interesting finding in the study was that the biliary duct plastic stent did not increase the risk of PEC. The patients with high leukocyte count before ERCP were predisposed to PEC. The correlations between white blood cell counts before ERCP and PEC were estimated using a binned scatterplot diagram. The results indicated that the risk of acute cholecystitis had a positive correlation with preoperative WBC. However, the association was not strong because the OR was low (OR = 1.1).

The present study, analyzed whether serum total bilirubin level and CBD diameter were risk factors for PEC. The result suggested no correlation between them. The result was different from those of previous studies. Lee et al. assessed the risk factors for acute cholecystitis after endoscopic CBD stone removal during a mean 18-month follow-up [9]. They reported that a serum total bilirubin level < 1.3 mg/dL and a CBD diameter < 11 mm at the time of endoscopic CBD stone removal were the risk factors for the development of PEC; the incidence of PEC was 17%. However, the follow-up time of their study on the occurrence of PEC was much longer than that in the present study.

The past history of acute pancreatitis was a risk factor of PEC in this study. The causes for previous acute pancreatitis in medical records for most patients were unclear and indefinite. It was difficult to explain why previous acute pancreatitis was associated with PEC. However, according to published endoscopic ultrasonography studies, a number of patients with past “idiopathic” acute pancreatitis might have suffered from acute pancreatitis due to microlithiasis and sludge.

**Fig. 4:**
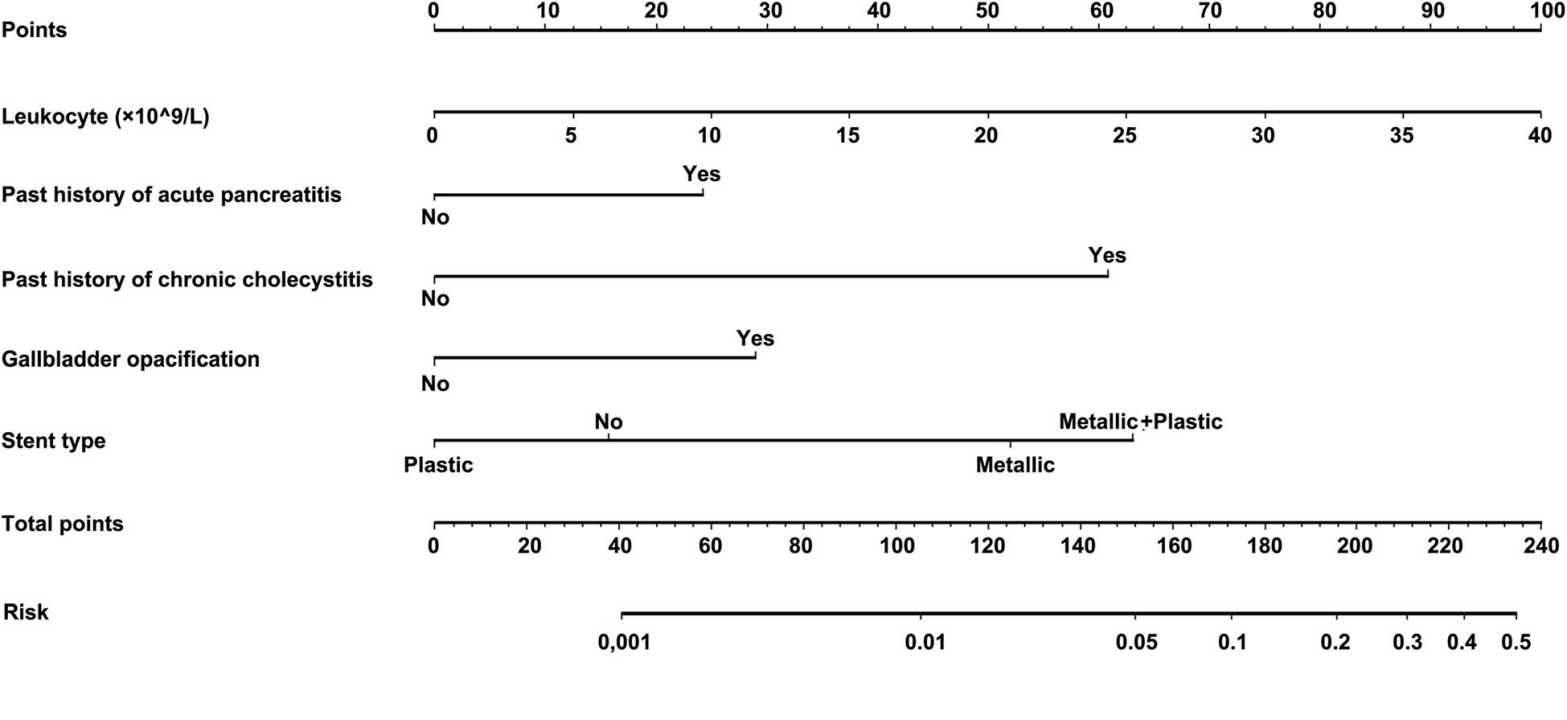
The Nomogram to predict the risk of post-ERCP cholecystitis. The behavioral variables are presented in rows 2–6, and points for each variable correspond to the scale in row 1. The points of five variables are added to the total points presented on the scale in row 7, which corresponds to the risk predictor of post-ERCP acute cholecystitis within 2 weeks in rows 8

In the present study, a practical nomogram was established to predict PEC with a good sensitivity and specificity (Fig. 4). According to the ROC of multivariate model, the AUC was 0.85 (95% CI 0.80–0.91), and the sensitivity and specificity were 82.3% and 73.3%, respectively. The nomogram revealed a good concordance and a good predictive ability for PEC. The present study reported the first nomogram for predicting PEC. External validation of this nomogram is needed in further studies.

Identifying the risk factors related to PEC is important for taking precautions to reduce the occurrence of PEC. When patients with these risk factors undergo ERCP, prophylactic measures should be taken to prevent PEC. Endoscopic gallbladder drainage, as a safe and efficacious internal drainage, improved patient pain and decreased the likelihood of the drain being dislodged [10]. Therefore, endoscopic gallbladder drainage has been used for elderly patients with multiple comorbidities at high risk for cholecystectomy to decompress the gallbladder as a temporary measure prior to surgery or as the definitive treatment [10–13]. Briefly, patients at high risk of PEC may undergo drainage with an endoscopically placed nasocholecystic tube or plastic stents.

The present study had several limitations. First, it was a single-center retrospective study with a possibility of accumulation of inappropriate data. Moreover, cholecystitis was not classified as acalculous or calculous because of the small number of patients with PEC. Prospective studies should be performed to further establish the risk factors for PEC.

## Conclusions

A history of acute pancreatitis, history of chronic cholecystitis, gallbladder opacification, biliary metal stent placement, and high leukocyte counts before ERCP were established as potential risk factors for the occurrence of PEC within 2 weeks by univariate and multivariate analyses. When patients with these risk factors undergo ERCP, prophylactic measures should be taken to prevent PEC.

## Abbreviations

AUC: Area under the curve
CBD: Common bile duct
CI: Confidence interval
ENBD: Nasobiliary drainage
ERCP: Endoscopic retrograde cholangiopancreatography
PEC: Post-ERCP cholecystitis
PEP: Post-ERCP pancreatitis
ROC: Receiver operating characteristic curve

## References

[1] Kochar B, Akshintala VS, Afghani E, Elmunzer BJ, Kim KJ, Lennon AM, Khashab MA, Kalloo AN, Singh VK (2015). Incidence, severity, and mortality of post-ERCP pancreatitis: a systematic review by using randomized, controlled trials. Gastrointest Endosc.

[2] Cotton PB, Garrow DA, Gallagher J, Romagnuolo J (2009). Risk factors for complications after ERCP: a multivariate analysis of 11,497 procedures over 12 years. Gastrointest Endosc.

[3] Wang P, Li ZS, Liu F, Ren X, Lu NH, Fan ZN, Huang Q, Zhang X, He LP, Sun WS (2009). Risk factors for ERCP-related complications: a prospective multicenter study. Am J Gastroenterol.

[4] Freeman ML, Nelson DB, Sherman S, Haber GB, Herman ME, Dorsher PJ, Moore JP, Fennerty MB, Ryan ME, Shaw MJ (1996). Complications of endoscopic biliary sphincterotomy. N Engl J Med.

[5] Yokoe M, Hata J, Takada T, Strasberg SM, Asbun HJ, Wakabayashi G, Kozaka K, Endo I, Deziel DJ, Miura F (2018). Tokyo guidelines 2018: diagnostic criteria and severity grading of acute cholecystitis (with videos). J Hepatobiliary Pancreat Sci.

[6] Zhang Z (2016). Univariate description and bivariate statistical inference: the first step delving into data. Ann Transl Med.

[7] Zhang Z (2016). Variable selection with stepwise and best subset approaches. Ann Transl Med.

[8] Zhang Z, Kattan MW (2017). Drawing Nomograms with R: applications to categorical outcome and survival data. Ann Transl Med.

[9] Lee JK, Ryu JK, Park JK, Yoon WJ, Lee SH, Lee KH, Kim YT, Yoon YB (2006). Risk factors of acute cholecystitis after endoscopic common bile duct stone removal. World J Gastroenterol.

[10] Itoi T, Kawakami H, Katanuma A, Irisawa A, Sofuni A, Itokawa F, Tsuchiya T, Tanaka R, Umeda J, Ryozawa S (2015). Endoscopic nasogallbladder tube or stent placement in acute cholecystitis: a preliminary prospective randomized trial in Japan (with videos). Gastrointest Endosc.

[11] Kjaer DW, Kruse A, Funch-Jensen P (2007). Endoscopic gallbladder drainage of patients with acute cholecystitis. Endoscopy.

[12] Mutignani M, Iacopini F, Perri V, Familiari P, Tringali A, Spada C, Ingrosso M, Costamagna G (2009). Endoscopic gallbladder drainage for acute cholecystitis: technical and clinical results. Endoscopy.

[13] Widmer J, Alvarez P, Sharaiha RZ, Gossain S, Kedia P, Sarkaria S, Sethi A, Turner BG, Millman J, Lieberman M (2015). Endoscopic gallbladder drainage for acute Cholecystitis. Clin Endosc.

